# Association of the *DYX1C1* Dyslexia Susceptibility Gene with Orthography in the Chinese Population

**DOI:** 10.1371/journal.pone.0042969

**Published:** 2012-09-13

**Authors:** Yuping Zhang, Jun Li, Twila Tardif, Margit Burmeister, Sandra M. Villafuerte, Catherine McBride-Chang, Hong Li, Bingjie Shi, Weilan Liang, Zhixiang Zhang, Hua Shu

**Affiliations:** 1 State Key Laboratory of Cognitive Neuroscience and Learning, Beijing Normal University, Beijing, China; 2 Department of Psychology, University of Michigan, Ann Arbor, Michigan, United States of America; 3 Molecular and Behavioral Neuroscience Institute, University of Michigan, Ann Arbor, Michigan, United States of America; 4 Department of Psychiatry, University of Michigan, Ann Arbor, Michigan, United States of America; 5 Department of Human Genetics, University of Michigan, Ann Arbor, Michigan, United States of America; 6 Department of Psychology, The Chinese University of Hong Kong, Hong Kong, China; 7 Department of Psychology, Beijing Normal University, Beijing, China; 8 Peking University First Hospital, Beijing, China; National University of Singapore, Singapore

## Abstract

Several independent studies have supported the association of *DYX1C1* with dyslexia, but its role in general reading development remains unclear. Here, we investigated the contribution of this gene to reading, with a focus on orthographic skills, in a sample of 284 unrelated Chinese children aged 5 to 11 years who were participating in the Chinese Longitudinal Study of Reading Development. We tested this association using a quantitative approach for Chinese character reading, Chinese character dictation, orthographic judgment, and visual skills. Significant or marginally significant associations were observed at the marker rs11629841 with children's orthographic judgments at ages 7 and 8 years (all *P* values<0.020). Significant associations with Chinese character dictation (all *P* values<0.013) were also observed for this single-nucleotide polymorphism (SNP) at ages 9, 10, and 11 years. Further analyses revealed that the association with orthographic skills was specific to the processing of specific components of characters (*P* values<0.046). No association was found at either SNP of rs3743205 or rs57809907. Our findings suggest that *DYX1C1* influences reading development in the general Chinese population and supports a universal effect of this gene.

## Introduction

Dyslexia (reading disability), which affects 5–10% of school-aged children across populations, is a specific impairment in learning to read that is independent of educational opportunities and general cognitive abilities [1], [2], [3]. Dyslexia is generally identified by gross difficulties in reading and spelling [4], [5], [6] and has moderate-to-high heritability, ranging from 43–73% for reading to 55–75% for spelling [7], [8], [9]. Although the etiology for dyslexia is not clear, behavioral studies have suggested that there are several cognitive factors underlying dyslexia, such as orthographic skills [4], [10], [11], [12]. Indeed, the heritability of orthographic skills, or the ability to recognize specific letter patterns (orthography) of whole words, has been reported to range from 20–67% [7], [8], [12]. Recent molecular genetic studies have revealed several susceptibility candidates for dyslexia, including variants in the *DYX1C1* gene, which is one of the most robustly reproduced findings [4], [5]. However, only a few studies have reported a significant association between *DYX1C1* and orthographic skills [13], [14].

*DYX1C1* (dyslexia susceptibility 1 candidate 1) was first identified as a candidate gene for dyslexia susceptibility in a Finnish family transmitting a chromosome translocation [t(2; 15)(q11; q21)] that segregated with dyslexia [15]. Subsequently, multiple studies have supported the association between *DYX1C1* and dyslexia, although not all [13], [14], [16]–[26]. The most widely replicated SNPs are rs3743205 (-3G/A) and rs57809907 (1249G/T) [13], [14], [15], [17], [20], [21]. The rs3743205 SNP has been found to be associated with several phenotypes, including spelling, with the “G” allele specified as the risk allele, and the common haplotype rs3743205/rs57809907 (G/G) associated with dyslexia [17]. The SNP rs57809907 and the haplotype rs3743205/rs57809907 (G/G) have also been reported to be associated with a measure of orthographic coding choice (OC-choice), with the “G” allele rs57809907, in particular, with poor OC-choice performance in individuals from 264 nuclear families with dyslexia [14]. Nonetheless, conflicting results have also been found for both rs3743205 and rs57809907, with the opposite allelic association with dyslexia [15], [21]. Moreover, recent studies have also supported the role of *DYX1C1* in normal reading variations, albeit with different SNPs (e.g., rs17819126). In these studies [16], [18], as in the present study, children with dyslexia were neither specifically selected for, nor were they screened out, and thus they are assumed to represent the low tail of the normal distribution of reading ability in the population. Although *DYX1C1* has been reproducibly reported to be significantly associated with dyslexia, there are few studies of its association with orthography [13], [14], a phenotype that varies between languages. In alphabetic languages (e.g., English), words consist of letter strings, which directly represent sounds, and orthographic coding can be measured by tasks such as OC-choice, which taps the ability to recognize orthographic representations of whole words and retrieve appropriate phonological representations from a mental lexicon [14]. In contrast, Chinese writing is striking in its visual complexity and its more abstract relation between the visual representation of a word and its pronunciation. All characters are square shapes comprised of different components; a component usually involves a fixed internal structure and a legal position within a character [10], [27], [28]. Knowledge of orthography is developed gradually over the primary school years and beyond [28], [29], [30]. Indeed, with reading experience, the internal structures of Chinese characters are fairly predictable [30]. Thus, orthography skills in Chinese are based on two primary aspects [28], [31], [32]: the ability to master the rules in combining different components into characters [27] and awareness of internal structure and positions of components. However, to our knowledge, only one recent study has investigated the genetic association between *DYX1C1* variants and dyslexia in the Chinese population [13]. Specifically, Lim et al. [13] genotyped eight SNPs for 393 individuals from 131 Chinese families with dyslexia and reported a significant association of rs3743205, but not rs57809907, to several phenotypes of reading, including orthographic skills.

Here we report data examining the association of polymorphisms in *DYX1C1* with reading, spelling, and orthographic skills in a general population of Chinese children. Since the present data is part of a large longitudinal study, we were able to test this association in relation to children's reading development over time. We were especially interested in the association between *DYX1C1* and orthographic skills, including awareness of internal structures and positions of components, as children go through the stages of becoming fluent readers. In contrast to reading, spelling requires the retrieval of sound to print correspondences and, therefore, encompasses a higher level of orthographic involvement [33], [34]. To rule out the possibility that basic visual skills play a major role in the *DYX1C1*-orthography relationship, we also examined the association between *DYX1C1* and visual skills. Thus, in our study, four quantitative traits were measured at critical ages (between 5 and 11 years of age) during reading development: a) Chinese character reading, b) Chinese character dictation (spelling), c) visual-related skills, and d) orthographic judgment. We examined three SNPs that were previously found to be associated with dyslexia in previous studies [13], [14], [15], [17], [20], [21], including the two SNPs (rs3743205 and rs57809907) originally reported by Taipale et al. [15] and replicated by Wigg et al. and Scerri et al. [14], [17] and another SNP (rs11629841) that was also reported by Wigg et al. [17].

## Methods

### Subjects

We used data from a general population cohort of 284 unrelated children who were recruited from maternal-child health care clinics to develop the Chinese Communicative Development Inventory (CCDI) in 2000 [35] and were demographically representative of the city of Beijing. All of these children were tested annually on a variety of tasks as part of a large longitudinal study and all met the following inclusion criteria: native Mandarin speakers, no known intellectual disability, and a nonverbal IQ within normal limits as assessed by the Raven's Standard Progressive Matrices [36] (i.e., a score ≥16^th^ percentile, corresponding to approximately ≥−1 SD from the population average of the 50^th^ percentile). Informed written consent was obtained from the parents of all children. Ethical approval for the present study was obtained from the Institutional Review Board (IRB) of Beijing Normal University Imaging Center for Brain Research, the State Key Laboratory of Cognitive Neuroscience and Learning, and from the Health Sciences and Behavioral Sciences Institutional Review Boards (IRB-HSBS) of the University of Michigan.

### Phenotype measures

The selection of tasks for the present study was based on the criterion that each had been administered for at least three years (except for the visual-spatial relationship subtest) and appeared to make use of some visual or orthographic skills. These included tests of single Chinese character reading and dictation, visual-related skills, and orthographic awareness.

#### Chinese character reading

This test was administered when children were aged 7, 8, 9, and 10 years old. This single character reading test has been successfully used in previous studies [28], [37], [38] and is comparable with tests of single word reading commonly used in genetic association studies of dyslexia for English and other languages [6]. During the test, children were asked to read a list of characters of increasing difficulty at their own pace and were allowed self-corrections and guessing. The final score for this test was the total number of correctly pronounced characters. A simple version consisting of 100 single Chinese characters was used for children 7 years of age, while another more difficult version consisting of 150 single characters was used for children older than age 8.

#### Chinese character dictation

This test was used to measure children's spelling ability at from ages 9 to 11 years. Children were required to write down target characters that were orally presented. Characters were all familiar and presented within the context of two- to four-character words. All target characters were listed in order of increasing difficulty. The final score of this dictation test was the total number of characters correctly written down. Children were tested on 32 items at ages 9 and 10 and on 40 items at age 11. A similar task has been successfully used in a previous study examining children's spelling ability [39].

#### Visual-related skills

Three tests of visual-related skills were used in this study: a) the Visual-Spatial Relationships test, b) Visual Matching, and c) Cross Out. Each of these three tests has been successfully used in other studies of Chinese children [28], [40].

#### Visual-spatial skill

The visual-spatial relationships subtest from the Gardner's [41] Test of Visual-Perceptual Skills (non-motor) Revised was used to assess visual-spatial skill. This test consisted of one practice item and 16 test items, and each trial consisted of five black-and-white line drawings. Children were asked to distinguish which of the drawings was oriented differently from the others. The final score of this test was the total number of correct responses. Due to subsequent ceiling effects with older children, this test was used only when children were 5 years old.

#### Processing speed

Two subtests from the Woodcock-Johnson Test of Cognitive Ability [42] were used to assess processing speed. We conceptualized both of these tasks as involving visual manipulations, since each requires visual analysis and identification. The Visual Matching test measures the ability to quickly locate and circle the 2 identical digits out of 6 in each row (e.g., 8 9 5 2 9 7) within 3 minutes. The whole test consists of 60 rows, and one practice trial was given before the formal test. The task advanced in difficulty from single-digit numbers to triple-digit numbers.

The Cross Out test measures the ability to quickly scan and compare visual information. In this task, children were asked to mark 5 out of 20 drawings in a row that were identical to the target drawing at the left end of the row in 3 minutes and the whole test was comprised of 30 rows. One practice trial was given before the formal test. For both subtests, correct responses were tallied as 1 point per row. These two tests were administered at ages 6, 7, and 8 years old.

#### Orthographic judgment

This test is aimed at measuring orthographic awareness and has been successfully used in a previous study examining predictors of reading in Chinese children [28]. In this test, children were asked to decide whether the visually presented items were real characters or not in a list of 70 items that included 40 test items and 30 real Chinese character fillers. Of the 40 test items, 10 were black-and-white drawings with no conventional stroke pattern used (Figure S1-A, Supporting Information) and 10 were ill-formed structure non-characters with real radicals in illegal positions (Figure S1-B, Supporting Information). Another 10 compound non-characters were ill-formed components with pseudo components or subcomponents from the Chinese writing system (Figure S1-C, Supporting Information). In addition, there were 10 well-formed structured pseudo-character items consisting of real components with components in their legal position (Figure S1-D, Supporting Information). The performance score was the number of items correctly rejected amongst the 40 test items. This test was used when the children were 6, 7, and 8 years old.

Descriptive statistics for all quantitative measures at each time point are presented in Table S1, with obvious increases with age observed in Visual Matching, Cross Out, orthographic judgment, Chinese character reading, and Chinese character dictation, with no floor or ceiling effects. Data for the Cross Out task for children at 7 years of age showed a poor distribution and was log-transformed to generate a more normal distribution. Table S2 presents the correlation coefficients between phenotypic measures and all significant correlations. The Chinese character reading and Chinese character dictation tests were strongly correlated across all test points (all correlation coefficients >0.63). Both Visual Matching and Cross out measured across children's test sessions from 6- to 8-year-old were significantly and moderately correlated with the Visual-Spatial Relationship test measured at age 5, providing support that these measures share the same visual construct. These visual-related tests and orthographic judgment tests were also found to correlate significantly with the reading measures.

### Genotyping

The three SNPs (rs3743205, rs11629841, and rs57809907) that showed significant associations in previous studies [15], [17] were genotyped using the MassArray system (Sequenom) with primers and probes shown in Table S3. The sample success rates for all three SNPs reached 100%. Four samples were genotyped twice and the reproducibility of the genotyping was 100%.

### Statistical analyses

The PLINK program version 1.07 [43] was used to carry out the Hardy-Weinberg Equilibrium (HWE) test of all three SNPs and to examine the association between SNPs or haplotypes and the quantitative measures. In line with previous studies [15], [18], additive models were considered and were evaluated against the null hypothesis of no association. As the three SNPs were low in linkage disequilibrium (LD) (Table S4, r^2^ ranged from 0.002 to 0.054), only the widely reported haplotype spanning rs57809907/rs3743205 [14], [15], [17] was analyzed in the present study. Linkage disequilibrium of the SNPs was estimated using Haploview Version 4.2 [44]. The SPSS 13.0 program was used to perform descriptive statistics and Pearson correlations. SNP association was performed with Chinese character reading and Chinese character dictation in the first step. SNPs that showed significant association in the first step were then further explored with visual-related measures and orthographic judgment. Finally, SNPs that showed significant association with orthographic judgments were then tested for associations with each of the four orthographic conditions. In all analyses, both age and sex were specified as covariates. We corrected for the number of SNPs, which yielded a significance level of 0.0167 after Bonferroni correction. Since all quantitative phenotypes were highly correlated (see Table S2), we did not correct for multiple phenotypes tested.

## Results

No deviation from HWE was found at any of these SNPs (Table S4). Allele frequencies for rs3743205 and rs57809907 were consistent with previous studies [15], [17], [18]. However, a minor allele frequency of 0.081 was observed for rs11629841, which is lower than that reported by Wigg et al. (MAF = 0.336) [17].

**Figure 1:**
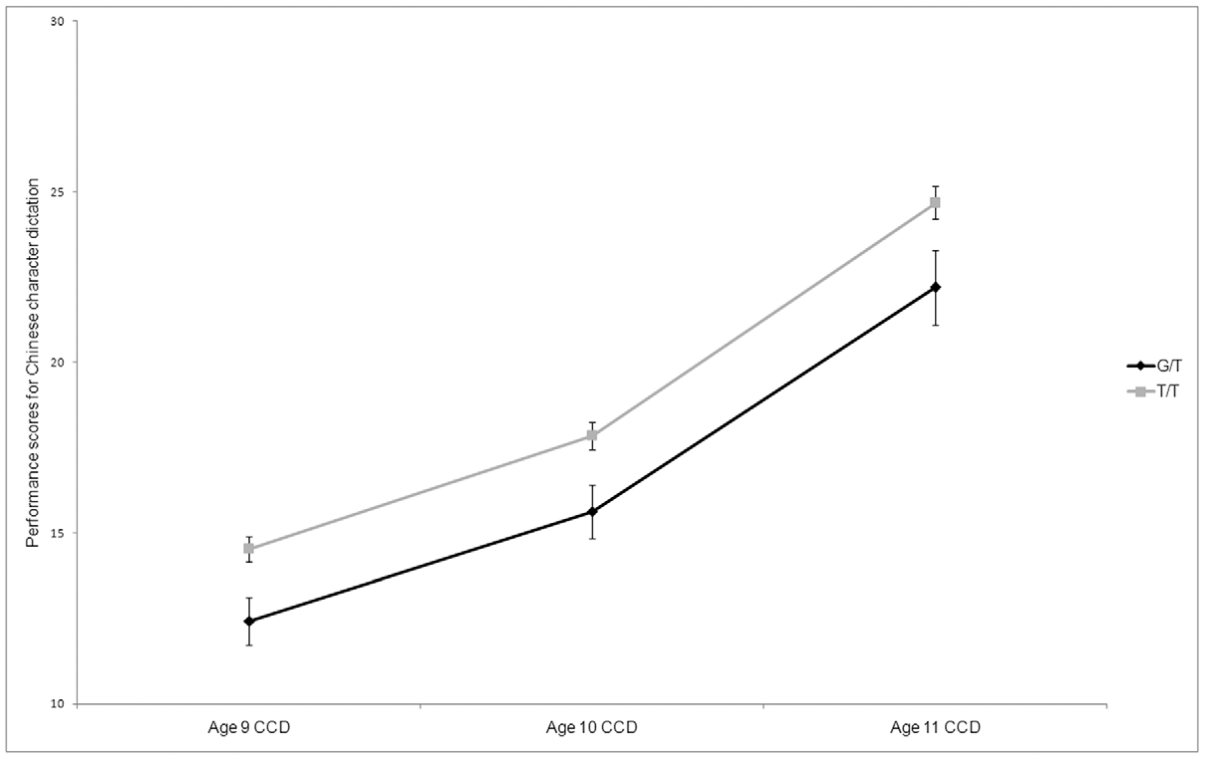
Performance of different genotypic groups of rs11629841 in Chinese character dictation. *N* = 0, 46, and 238 for the three genotypes of G/G, G/T, and T/T respectively. X-lab = test time (in years), Y-lab = performance scores for Chinese character dictation. CCD = Chinese character dictation.

**Figure 2:**
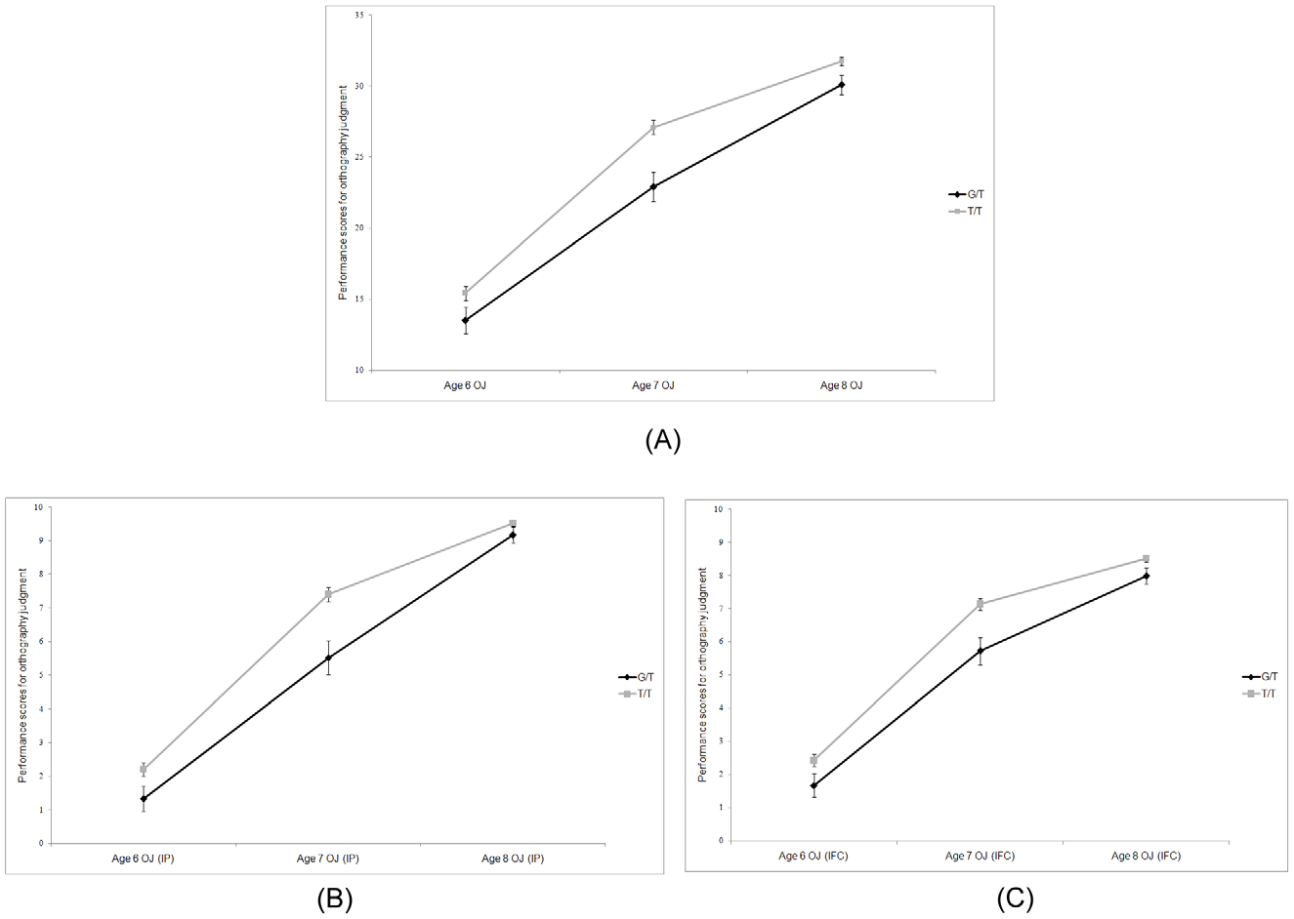
Performance of different genotypic groups of rs11629841 in orthographic judgment. Orthographic judgment (all four conditions combined) (A), illegal position (B), and ill-formed component (C). *N* = 0, 46, and 238 for the three genotypes of G/G, G/T, and T/T respectively. X-lab = test time (in years), Y-lab = performance scores for orthographic judgment. OJ = orthographic judgment, IP = illegal position, IFC = ill-formed component.

In the single-marker analyses, association signals were found between rs11629841 and Chinese character dictation at all three test points (*P* = 0.012, 0.009, and 0.013 for 9-, 10-, and 11-year-olds, respectively) and remained significant after correcting for multiple comparisons, with the minor allele “G” associated with poor performance (Table 1 and Figure 1). However, no significant association with this SNP was found for Chinese character reading (all *P* values>0.05). Further analyses with orthographic skills showed a significant or marginally significant association between the same SNP and orthographic judgment at ages 7 and 8 (*P* = 0.0003 and *P* = 0.0198, respectively), although only the results at age 7 remained significant after correcting for multiple comparisons, with the minor allele “G” significantly associated with poor performance (Table 2 and Figure 2-A). A similar trend was also found at age 6 (*P* = 0.109). No association was found between rs11629841 and visual-related skills for ages 5 to 8 (all *P* values>0. 05). Of the four conditions in the orthographic judgment test, a significant association was found for the condition of illegal position (Figure 2-B) at age 7 (*P* = 0.0002) and a similar association trend was found for the other two test points (*P* = 0.082 and *P* = 0.098 for ages 6 and 8, respectively). Significant or marginally significant associations were also found for the condition of ill-formed components (Figure 2-C) for two test points (*P* = 0.0004 and *P* = 0.029 for ages 7 and 8, respectively), and the same association trend was found at age 6 (*P* = 0.096). No association was found for the other two conditions for any of the three test points (all *P* values>0.05). In addition, no associations, even at the nominal level, were observed for the other two markers, rs3743205 and rs57809907 (all *P* values>0.10).

**Table 1:** Association of *DYC1C1* with reading and dictation.

	Reading								Dictation					
	Age 7 CCR		Age 8 CCR		Age 9 CCR		Age 10 CCR		Age 9 CCD		Age 10 CCD		Age 11 CCD	
	B	*P*	β	*P*	β	*P*	β	*P*	β	*P*	β	*P*	β	*P*
rs3743205	−1.66	0.543	0.29	0.936	−0.52	0.887	0.95	0.736	−0.09	0.933	−0.72	0.540	−0.16	0.909
rs11629841	−4.17	0.058	−5.61	0.059	−5.64	0.053	−3.80	0.093	−2.24	0.012**	−2.47	0.009**	−2.82	0.013**
rs57809907	−1.73	0.670	−0.86	0.875	1.67	0.756	1.56	0.707	−0.62	0.707	−1.45	0.405	−1.41	0.502

*Note.* CCR, Chinese character reading; CCD, Chinese character dictation.

* P<0.05,

** P<0.0167.

**Table 2:** Association of *DYC1C1* with visual and orthographic skills.

	rs11629841							
	Age 5		Age 6		Age 7		Age 8	
	β	*P*	β	*P*	β	*P*	β	*P*
Visual-related skills								
VSR	−0.60	0.306	NA	NA	NA	NA	NA	NA
VM	NA	NA	−0.35	0.683	−0.69	0.391	−1.49	0.089
VC	NA	NA	−0.20	0.664	−0.29	0.541	−0.55	0.295
Orthographic judgment								
OJ	NA	NA	−1.84	0.109	−4.40	0.0003**	−1.76	0.0189*
OJ (black-and-white drawings)	NA	NA	0.025	0.844	−0.17	0.067	−0.024	0.746
OJ (illegal position)	NA	NA	−0.84	0.082	−1.97	0.0002**	−0.39	0.098
OJ (ill-formed component)	NA	NA	−0.73	0.096	−1.50	0.0004**	−0.56	0.028*
OJ (pseudo-character)	NA	NA	−0.30	0.358	−0.75	0.103	−0.78	0.092

*Note.* VSR, the visual spatial relationship test; VM, Visual Matching; VC, Cross Out; OJ, orthographic judgment.

NA, Not available.

We also performed a haplotypic analysis for the two-marker combination, namely rs3743205/rs57809907. Similar haplotype frequencies were observed: G/C (0.938), G/T (0.041), T/C (0.013) and T/T (0.008), but neither the rare haplotype nor the common haplotype that has been previously reported [14], [15], [17], [21] to influence dyslexia was found in association with our sample (all *P* values>0.10).

## Discussion

We investigated the association between *DYX1C1* and reading skills in a general population of Chinese children followed from ages 5 to 11. The study is significant in its utilization of a longitudinal design in examining this association over multiple time points in relation to general reading abilities in the Han population. Support was found for an association between rs11629841 and Chinese character dictation, but not Chinese character reading, at all three test points (ages 9, 10, and 11 years), with the “G” allele associated with poor performance. Given that spelling ability is both dependent on and a reflection of orthographic skills [33], further analyses were done with orthographic skills. We found a significant association between the same SNP and orthographic judgment at two out of three test points (ages 7 and 8 years, but not 6 years), particularly with the two conditions that involved component processing of characters that were illegally positioned or ill-formed. An association for visual-related skills was also tested, but as expected, no association signal was observed between rs11629841 and basic visual skills throughout all ages tested (5 to 8 years). Together these results suggest a possible role for this SNP in the etiology of orthographic skills and, therefore, spelling skills.

The significant associations between rs11629841 and orthographic skills and spelling support its role in reading and are consistent with a study reporting biased transmission of the “G” allele of this SNP in a sample of 148 Caucasian families with dyslexia [17]. However, in another study of 247 parent-proband trios from the UK [23], Cope et al. failed to find any association between rs11629841 and dyslexia or varied reading measures, including spelling and orthography [23]. This discrepancy between our findings and those of Cope et al. [23] may be due to many factors.

First, the nature of orthography was different in our study, when compared with the English language study of Cope et al. [23]. Based on previous behavioral, patient, and brain studies, awareness of internal structure and position of components are the basic orthographic skills necessary for the Chinese language [10], [28], [30], [31], [32], [45], which are quite different from those for the English language. Moreover, different biological and neural mechanisms underlie the processing of these two languages [31], [32], [46]. Han et al. [31] reported a Chinese dysgraphic patient whose errors in the delayed copy task were mostly at the logographeme level, suggesting the component level of processing can be selectively impaired in the brain. Another study found significantly greater activation in the left middle fusiform gyrus (MFG), the Visual Word Form Area (VWFA) [32], during real and pseudo character processing compared to artificial characters, suggesting a crucial role of the VWFA in orthography or component processing of Chinese characters. Combined, we agree that these findings, together with our association results, imply a possible role of rs11629841 in the etiology of orthography. Nonetheless, given that the present study is the first of its kind to examine genetic association orthographic in Chinese, further research in this area that allows for functional analyses is required before a strong conclusion can be drawn.

Second, compared with the wide age range (6–17 years) in Cope et al.'s sample population, we assessed children's reading skills longitudinally with all children almost at the same ages in the present study and reported significant associations at specific ages during development.

Third, differences in the MAF between samples may also be a reason for a discrepancy in findings (8.1% in the present sample vs. about 32% in reported samples). Although the lower MAF may reduce the reliability of the present findings, this genuinely reflects the cases of different populations as shown by the MAF based on the Hapmap data, with about 35% in Caucasian, 17% in African American, but only 9% in Chinese, and 2% in Japanese.

Finally, other factors may also have contributed, such as different samples (families with dyslexia probands in Cope et al.'s study vs. general population in the present study), sex effects (with a ratio of about 2∶1 for male and female in samples of dyslexia vs. about 1∶1 in the general population). At this point, it is not possible to determine the precise reasons for the discrepancies. Nonetheless, the present results, together with those of Cope et al. [23] suggested several important directions for future research.

In contrast, SNP rs3743205 was not associated with any measure in this study, which is inconsistent with a recent dyslexia study of Hong Kong Chinese that reported a significant association with multiple reading skills, including orthographic skills [13]. There are several factors that may explain this discrepancy between the study with Hong Kong Chinese children and our study with Mandarin Chinese children. As stated earlier, the characteristics of the research samples (dyslexia vs. general sample; more heterogeneous Han population in our sample) may result in different findings. It is possible that this SNP (rs3743205) only influences reading skills in dyslexia since no association was found in two previous studies on general populations [16], [18]. In addition, the previous study focused on children learning to read traditional Chinese characters, whereas the present study included only children reading simplified characters. Since less information is provided by fewer strokes and simpler structure, mastering simplified Chinese used in Mainland China may require slightly different visual and orthographic analyses than those required for traditional Chinese characters [47]. Finally, the clear differences in educational practices and language environment (bilingual vs. monolingual) between the two cultures may also contribute to this discrepancy. Although a number of previous studies have discussed the role of rs3743205, its association with either orthographic skills or spelling abilities has only been found in two other studies [14], [17] while six other studies failed to find any association, even with dyslexia [16], [18], [19], [22], [23], [24]. The lack of association with rs3743205 does not preclude our findings with rs11629841 that supporting the association between *DYX1C1* and orthographic skills. It is possible that there is a third SNP, in high LD with both rs3743205 and rs11629841, that contributes to the association between *DYX1C1* and orthography.

We also failed to find an association between rs57809907 and any of our reading measures in this study, which is consistent with the study of Hong Kong Chinese dyslexic readers [13]. Several previous studies of dyslexia also failed to find any association with this SNP [18], [22], [23], [25], [26], although some studies on Caucasian populations that use an alphabetic language reported a positive association [14], [15], [19], [21]. As mentioned above, discrepancies between these studies may due to language differences, sex effects, and many other factors. It is also possible that this SNP is simply not associated with reading abilities in the Chinese population.

We are aware that the present study may have been potentially hampered by several limitations, for example, the small sample size, exploratory analysis of the *DYX1C1* effect in the Han population, and limited *DYX1C1* marker coverage. Although our study suggests that a longitudinal design may be advantageous in identifying additional causal alleles for reading phenotypes, further research using a larger sample of Chinese children is still needed in replicating these findings. Nonetheless, our findings are consistent with previous reports in supporting the role of the dyslexia candidate gene of *DYX1C1* in general reading variation [16], [18]. In particular, they provide new evidence on the associations between rs11629841 and orthographic skills and spelling in a non-European population.

## Supporting Information

Figure S1 **Examples of the orthographic judgment test, black-and-white drawing (A), illegal position (B), ill-formed component (C), and pseudo characters (D).**
(TIF)

Table S1 **Descriptive statistics of phenotype measures.** *The absolute scores were log-transformed for Age 7 cross out to achieve suitable skewness and kurtosis values.
(DOC)

Table S2 **Pearson correlations between phenotype measures.** VSR- the Visual-Spatial Relationship test, VM-Visual Matching, VC-Cross Out, OJ-orthographic judgment, CCR-Chinese character reading, CCD-Chinese character dictation. Correlation coefficients above 0.19 are significant at P<0.001, correlation coefficients above 0.15 are significant at p<0.01, and correlation coefficients above 0.12 are significant at p<0.05, *N* = 284.
(DOC)

Table S3 **Markers genotyped by MassArray system (sequenom) probes.**
(DOC)

Table S4 **Linkage disequilibrium. MAF, minor allele frequency.** D′ values are shown in the upper half of the table, and r^2^ values in the lower half of the table.
(DOC)
