# Supplementary figures and images for: Association of the DYX1C1 Dyslexia Susceptibility Gene with Orthography in the Chinese Population
Source: PLoS One. 2012 Sep 13;7(9):e42969. doi: 10.1371/journal.pone.0042969 (PMC3441603; doi:10.1371/journal.pone.0042969)

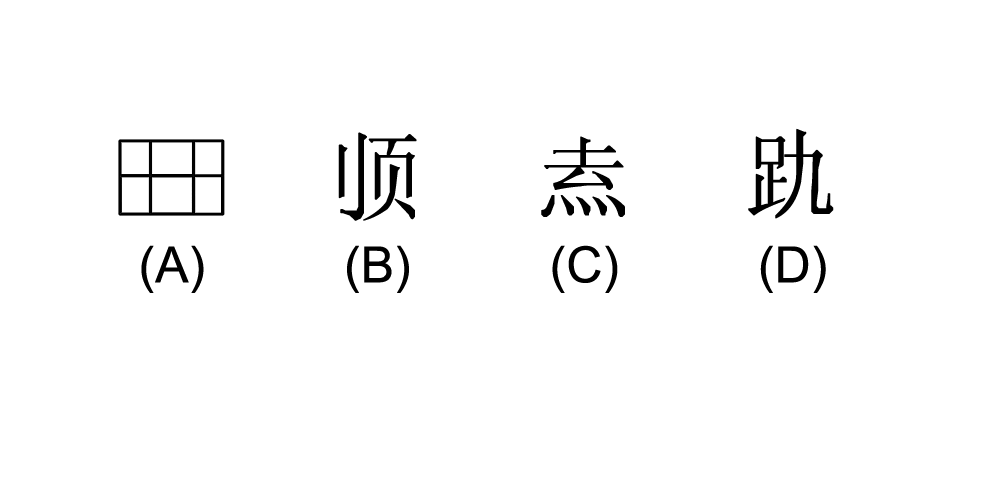

Supplement: Figure S1 — Examples of the orthographic judgment test, black-and-white drawing (A), illegal position (B), ill-formed component (C), and pseudo characters (D). (TIF) [file pone.0042969.s001.tif]
