# Supplementary material for: Association of the DYX1C1 Dyslexia Susceptibility Gene with Orthography in the Chinese Population
Source: PLoS One. 2012 Sep 13;7(9):e42969. doi: 10.1371/journal.pone.0042969 (PMC3441603; doi:10.1371/journal.pone.0042969)
Supplement: Table S1 — Descriptive statistics of phenotype measures. *The absolute scores were log-transformed for Age 7 cross out to achieve suitable skewness and kurtosis values. (DOC) [file pone.0042969.s002.doc]

Table S1

|  | N | Mean | SD | Range | Skewness | Kurtosis |
| --- | --- | --- | --- | --- | --- | --- |
| Age 5 Visual spatial relationship (VSR) | 284 | 11.45 | 3.68 | 1-16 | -1.03 | 0.48 |
| Age 6 Visual matching (VM) | 284 | 21.84 | 5.47 | 0-37 | -0.17 | 0.66 |
| Age 7 Visual matching | 284 | 29.55 | 5.07 | 13-43 | -0.01 | 0.62 |
| Age 8 Visual matching | 284 | 35.08 | 5.55 | 17-51 | -0.22 | 0.08 |
| Age 6 Cross out (VC) | 284 | 10.46 | 2.90 | 1-18 | -0.46 | 0.90 |
| Age 7 Cross out* | 284 | 15.18 | 3.10 | 7-31 | 0.15 | 1.79 |
| Age 8 Cross out | 284 | 17.89 | 3.35 | 9-28 | 0.10 | 0.09 |
| Age 6 Orthography judgment (OJ) | 284 | 15.12 | 7.48 | 6-38 | 1.50 | 1.26 |
| Age 7 Orthography judgment | 284 | 26.42 | 7.67 | 9-40 | -0.33 | -0.83 |
| Age 8 Orthography judgment | 284 | 31.48 | 4.66 | 12-40 | -0.94 | 1.86 |
| Age 7 Chinese character reading (CCR) | 284 | 56.31 | 14.42 | 8-89 | 0.02 | -0.08 |
| Age 8 Chinese character reading | 284 | 89.20 | 19.33 | 36-140 | 0.20 | -0.18 |
| Age 9 Chinese character reading | 284 | 111.58 | 18.61 | 39-145 | -0.85 | 0.96 |
| Age 10 Chinese character reading | 284 | 123.43 | 14.47 | 63-149 | -0.70 | 0.65 |
| Age 9 Chinese character dictation (CCD) | 284 | 14.19 | 5.59 | 2-30 | 0.24 | -0.50 |
| Age 10 Chinese character dictation | 284 | 17.49 | 6.03 | 0-32 | -0.09 | -0.32 |
| Age 11 Chinese character dictation | 284 | 24.28 | 7.23 | 4-39 | -0.35 | -0.36 |
| Raven’s Progressive Matrices (percentiles) | 284 | 71.81 |  | 16-95 |  |  |
