# Supplementary material for: Association of the DYX1C1 Dyslexia Susceptibility Gene with Orthography in the Chinese Population
Source: PLoS One. 2012 Sep 13;7(9):e42969. doi: 10.1371/journal.pone.0042969 (PMC3441603; doi:10.1371/journal.pone.0042969)
Supplement: Table S2 — Pearson correlations between phenotype measures. VSR- the Visual-Spatial Relationship test, VM-Visual Matching, VC-Cross Out, OJ-orthographic judgment, CCR-Chinese character reading, CCD-Chinese character dictation. Correlation coefficients above 0.19 are significant at P<0.001, correlation coefficients above 0.15 are significant at p<0.01, and correlation coefficients above 0.12 are significant at p<0.05, N = 284. (DOC) [file pone.0042969.s003.doc]

Table S2

|  |  | 1 | 2 | 3 | 4 | 5 | 6 | 7 | 8 | 9 | 10 | 11 | 12 | 13 | 14 | 15 | 16 | 17 |
| --- | --- | --- | --- | --- | --- | --- | --- | --- | --- | --- | --- | --- | --- | --- | --- | --- | --- | --- |
| 1 | Age 5 VSR | -- |  |  |  |  |  |  |  |  |  |  |  |  |  |  |  |  |
| 2 | Age 6 VM | 0.35 | -- |  |  |  |  |  |  |  |  |  |  |  |  |  |  |  |
| 3 | Age 7 VM | 0.31 | 0.61 | -- |  |  |  |  |  |  |  |  |  |  |  |  |  |  |
| 4 | Age 8 VM | 0.35 | 0.62 | 0.70 | -- |  |  |  |  |  |  |  |  |  |  |  |  |  |
| 5 | Age 6 VC | 0.30 | 0.58 | 0.43 | 0.42 | -- |  |  |  |  |  |  |  |  |  |  |  |  |
| 6 | Age 7 VC | 0.33 | 0.47 | 0.60 | 0.52 | 0.42 | -- |  |  |  |  |  |  |  |  |  |  |  |
| 7 | Age 8 VC | 0.40 | 0.51 | 0.57 | 0.67 | 0.47 | 0.54 | -- |  |  |  |  |  |  |  |  |  |  |
| 8 | Age 6 OJ | 0.14 | 0.31 | 0.16 | 0.24 | 0.23 | 0.22 | 0.15 | -- |  |  |  |  |  |  |  |  |  |
| 9 | Age 7 OJ | 0.25 | 0.38 | 0.30 | 0.37 | 0.27 | 0.28 | 0.25 | 0.36 | -- |  |  |  |  |  |  |  |  |
| 10 | Age 8 OJ | 0.28 | 0.34 | 0.26 | 0.30 | 0.21 | 0.22 | 0.28 | 0.15 | 0.53 | -- |  |  |  |  |  |  |  |
| 11 | Age 7 CCR | 0.32 | 0.48 | 0.45 | 0.48 | 0.35 | 0.43 | 0.42 | 0.43 | 0.52 | 0.44 | -- |  |  |  |  |  |  |
| 12 | Age 8 CCR | 0.32 | 0.47 | 0.44 | 0.45 | 0.33 | 0.41 | 0.40 | 0.39 | 0.53 | 0.47 | 0.88 | -- |  |  |  |  |  |
| 13 | Age 9 CCR | 0.30 | 0.44 | 0.45 | 0.49 | 0.29 | 0.40 | 0.42 | 0.24 | 0.49 | 0.50 | 0.79 | 0.88 | -- |  |  |  |  |
| 14 | Age 10 CCR | 0.31 | 0.40 | 0.44 | 0.46 | 0.29 | 0.40 | 0.41 | 0.24 | 0.50 | 0.47 | 0.76 | 0.87 | 0.91 | -- |  |  |  |
| 15 | Age 9 CCD | 0.29 | 0.39 | 0.37 | 0.43 | 0.25 | 0.35 | 0.35 | 0.29 | 0.51 | 0.42 | 0.71 | 0.77 | 0.76 | 0.76 | -- |  |  |
| 16 | Age 10 CCD | 0.27 | 0.37 | 0.39 | 0.45 | 0.25 | 0.38 | 0.37 | 0.26 | 0.47 | 0.38 | 0.68 | 0.72 | 0.76 | 0.77 | 0.81 | -- |  |
| 17 | Age 11 CCD | 0.30 | 0.36 | 0.36 | 0.44 | 0.24 | 0.35 | 0.36 | 0.23 | 0.44 | 0.37 | 0.63 | 0.69 | 0.74 | 0.75 | 0.79 | 0.86 | -- |
