# Supplementary material for: Association of the DYX1C1 Dyslexia Susceptibility Gene with Orthography in the Chinese Population
Source: PLoS One. 2012 Sep 13;7(9):e42969. doi: 10.1371/journal.pone.0042969 (PMC3441603; doi:10.1371/journal.pone.0042969)
Supplement: Table S3 — Markers genotyped by MassArray system (sequenom) probes. (DOC) [file pone.0042969.s004.doc]

Table S3

| Marker | Forward Primers | Reverse Primers | Probes |
| --- | --- | --- | --- |
| rs3743205 | ACGTTGGATGAAGCAGGCGCAAGAAGCAAC | ACGTTGGATGGTAATCGCTAACCTGAAGAG | ATCCGCTCCCGTTGCTACC |
| rs11629841 | ACGTTGGATGAGGGCATGTGTATTCACTG | ACGTTGGATGGGTATGAGTTGCTAGTTGTC | CTAGTTGTCTATTTAATGCCAT |
| rs57809907 | ACGTTGGATGATGCGAGAAGATTCGGAATG | ACGTTGGATGCTTAGTTACTTCTAATAGTC | TCGGAATGTAATTCAAGGAACA |
