# Supplementary material for: Association of the DYX1C1 Dyslexia Susceptibility Gene with Orthography in the Chinese Population
Source: PLoS One. 2012 Sep 13;7(9):e42969. doi: 10.1371/journal.pone.0042969 (PMC3441603; doi:10.1371/journal.pone.0042969)
Supplement: Table S4 — Linkage disequilibrium. MAF, minor allele frequency. D′ values are shown in the upper half of the table, and r2 values in the lower half of the table. (DOC) [file pone.0042969.s005.doc]

Table S4

| SNP | Minor/Major | Genotype |  | MAF | rs3743205 | rs11629841 | rs57809907 |
| --- | --- | --- | --- | --- | --- | --- | --- |
| rs3743205 | T/C | 0/28/256 |  | 0.049 | -- | 1 | 0.359 |
| rs11629841 | G/T | 0/46/238 |  | 0.081 | 0.005 | -- | 1 |
| rs57809907 | T/G | 0/12/272 |  | 0.021 | 0.054 | 0.002 | -- |
